# Compromised Behavior and Gamma Power During Working Memory in Cognitively Healthy Individuals With Abnormal CSF Amyloid/Tau

**DOI:** 10.3389/fnagi.2020.574214

**Published:** 2020-10-14

**Authors:** Roger Rochart, Quanying Liu, Alfred N. Fonteh, Michael G. Harrington, Xianghong Arakaki

**Affiliations:** ^1^Neurosciences, Huntington Medical Research Institutes, Pasadena, CA, United States; ^2^Department of Biomedical Engineering, Southern University of Science and Technology (SUSTech), Shenzhen, China

**Keywords:** EEG, behavioral performance, gamma, working memory, high risk of Alzheimer’s disease, CH-NAT, CH-PAT

## Abstract

Research shows that gamma activity changes in Alzheimer’s disease (AD), revealing synaptic pathology and potential therapeutic applications. We aim to explore whether cognitive challenge combined with quantitative EEG (qEEG) can unmask abnormal gamma frequency power in healthy individuals at high risk of developing AD. We analyzed low (30–50 Hz) and high gamma (50–80 Hz) power over six brain regions at EEG sensor level (frontal/central/parietal/left temporal/right temporal/occipital) in a dataset collected from an aging cohort during N-back working memory (WM) testing at two different load conditions (*N* = 0 or 2). Cognitively healthy (CH) study participants (≥60 years old) of both sexes were divided into two subgroups: **n**ormal **a**myloid/**t**au ratios (CH-NAT, *n* = 10) or **p**athological **a**myloid/**t**au (CH-PAT, *n* = 14) in cerebrospinal fluid (CSF). During low load (0-back) challenge, low gamma is higher in CH-PATs than CH-NATs over frontal and central regions (*p* = 0.014∼0.032, effect size (Cohen’s *d*) = 0.95∼1.11). However, during high load (2-back) challenge, low gamma is lower in CH-PATs compared to CH-NATs over the left temporal region (*p* = 0.045, Cohen’s *d* = −0.96), and high gamma is lower over the parietal region (*p* = 0.035, Cohen’s *d* = −1.02). Overall, our studies show a medium to large negative effect size across the scalp (Cohen’s *d* = −0.51∼−1.02). In addition, low gamma during 2-back is positively correlated with 0-back accuracy over all regions except the occipital region only in CH-NATs (*r* = 0.69∼0.77, *p* = 0.0098∼0.027); high gamma during 2-back correlated positively with 0-back accuracy over all regions in CH-NATs (*r* = 0.68∼0.78, *p* = 0.007∼0.030); high gamma during 2-back negatively correlated with 0-back response time over parietal, right temporal, and occipital regions in CH-NATs (*r* = −0.70∼−0.66, *p* = 0.025∼0.037). We interpret these preliminary results to show: (1) gamma power is compromised in AD-biomarker positive individuals, who are otherwise cognitively healthy (CH-PATs); (2) gamma is associated with WM performance in normal aging (CH-NATs) (most significantly in the frontoparietal region). Our pilot findings encourage further investigations in combining cognitive challenges and qEEG in developing neurophysiology-based markers for identifying individuals in the prodromal stage, to help improving our understanding of AD pathophysiology and the contributions of low- and high-frequency gamma oscillations in cognitive functions.

## Introduction

### Working Memory (WM) and Alzheimer’s Disease (AD)

Working memory (WM) refers to a core executive function that maintains and manipulates short term information for learning and reasoning (Baddeley, 1998a). Working memory processes are mediated by the frontal cortex and play a critical role in cognitive processing in everyday functions (Baddeley, 1998a, b). Alzheimer’s Disease (AD) is a neurodegenerative condition characterized by the accumulation of beta-amyloid plaques and neurofibrillary tangles from hyperphosphorylated-tau that disrupt synapses and lead to cognitive deficits (Rajmohan and Reddy, 2017; Sperling et al., 2019). Studies have demonstrated that AD patients have working memory (WM) dysfunction (Salthouse and Ferrer-Caja, 2003; Salthouse, 2003; Sperling et al., 2011). In addition, previous studies have shown that working memory begins to decline by 18 years of age, and EEG alpha power during WM processing is dysfunctional beginning in early AD (Rabinowitz et al., 1982; Craik et al., 1987; Craik and Dirkx, 1992; Salthouse, 2003; Arakaki et al., 2019).

Pre-symptomatic Alzheimer’s Disease (AD) is an active research area because AD treatment may need to start before damage becomes symptomatic. In our studies, we focused on cognitively healthy (CH) participants aged 60 and older with either a pathological (CH-PAT) or normal (CH-NAT) β_42_-amyloid/tau ratio within the cerebrospinal fluid (CSF) (Harrington et al., 2013). Our longitudinal study showed that CH-PATs had a significantly higher risk of cognitive decline to mild cognitive impairment (MCI) or AD compared to CH-NATs over four years (Wilder et al., 2018; Harrington et al., 2019). We also found CH-PATs present hyper-excitability during low load WM challenge shown by alpha event-related desynchronization (ERD) during quantitative EEG (qEEG) recordings (Arakaki et al., 2019).

### Gamma and AD

Studies have shown that alpha and gamma are associated during WM processing (Harrington et al., 2013; Arakaki et al., 2019). However, we do not know the role gamma power plays in WM processing in CH-PATs. The objective of this exploratory study was to evaluate changes in gamma activity in CH-PATs compared to CH-NATs using qEEG during the low load and high load N-back WM paradigm. We hypothesized that gamma power during high load N-back working memory testing in CH-PAT individuals is lower than in healthy aging and that gamma power correlates with N-back performance.

Gamma oscillations can be detected by qEEG and are modulated by both sensory input (i.e., stimulus) or internal regulatory mechanisms, and include two classes: high gamma frequency (>50 Hz) and low gamma frequency (30–50 Hz) (Jia and Kohn, 2011). Previous studies demonstrate an increase in both high and low gamma activity in healthy controls during verbal and non-verbal memory tasks (Tallon-Baudry et al., 1998; Sederberg et al., 2007; Palop and Mucke, 2016). Also, individuals formally diagnosed with AD show a significant reduction in spontaneous global gamma power activity compared to cognitively healthy individuals (Herrmann and Demiralp, 2005), suggesting a link between gamma activity and cognitive decline. Data from a mouse model of AD showed decreased gamma before the cognitive decline and photo-stimulation of modified cells with blue light at 40 Hz resulted in a significant increase in Aβ_42_ clearance and p-tau reduction in CA1 of the hippocampus (Iaccarino et al., 2016). Also, human studies have shown that gamma power is highly positively correlated with memory retrieval success and modulating accuracy (Stevenson et al., 2018) and gamma activity in epileptic patients increased with working memory load (Howard et al., 2003; van Vugt et al., 2010). These studies show that gamma activity plays a significant role in synaptic health and core executive functions such as working memory.

Synaptic dysfunctions reported in early AD have been detected by qEEG (Nava-Mesa et al., 2014; Babiloni et al., 2016c, 2020). Several studies have explored qEEG in relation to gamma activity. For example, when baseline power is set as a response measure for bars moving in random patterns on a screen, only bands within the low gamma region (35–45 Hz) show a significant change in response to the stimulus and an increase in spectral power (Lutzenberger et al., 1995). Furthermore, Tallon-Baudry et al. (1998) and Tallon-Baudry and Bertrand (1999) observed evoked gamma 90 ms after stimulus presentation and induced gamma 280 ms after stimulus presentation. This presence of both evoked and induced gamma power suggests a temporal domain for gamma activity in response to the regular visual stimulus. These studies demonstrate that qEEG is a powerful tool that can be used to examine changes in gamma power with high temporal resolution in high-risk individuals. In addition, it has been shown that healthy older adults and those with MCI lack modulation and have reduced gamma power, respectively, compared to healthy younger adults (Missonnier et al., 2004; Barr et al., 2014). Therefore, we propose that high-risk individuals may also show compromised gamma activity within cortical regions. Since little is known about CH-PAT individuals, qEEG can offer a non-invasive approach to detect early synaptic dysfunctions in the AD progression spectrum. This method has the potential to be complementary to current AD spectrum diagnosis [AT(N)] (Jack et al., 2018).

## Materials and Methods

### Participants

The Huntington Medical Research Institutes (HMRI) Institutional Review Board (IRB) has approved this study (Quorum IRB, Seattle, Study # 27197). All participants have signed informed consent forms.

We explored gamma power in our published dataset of 24 cognitively healthy participants whose ages ranged from 60 to 100 years for the pilot study (Arakaki et al., 2019). Briefly, an equal number of participants from each socioeconomic class was coded for the investigator to remain unbiased. Assessments included collection of demographic data, physical exam, blood work, disease severity and disability scales, and CSF amyloid/tau measurements (Harrington et al., 2013). Participants with any cognitive impairment, i.e., global clinical dementia rating scale (CDR) scores > 0, were excluded. Only participants who had Uniform Data Set-3 format examination with no classifiable psychiatric or neurological disorder were diagnosed as CH and enrolled in this study after a 5 h comprehensive neuropsychological battery in which testing was performed independent/blind to the Aβ_42_ and tau classifications. We test the cognitive domains of memory, executive function, language, attention, and visuospatial orientation. All data were normalized to age, sex, and normative education tables (Harrington et al., 2013). These formal neuropsychometric data were combined with clinical dementia rating, Montreal Cognitive Assessment, Mini-Mental State Examination, as described (Harrington et al., 2013). Participants were then classified, depending on individual CSF Aβ/tau ratios, as either normal (CH-NATs) or pathological (CH-PATs), compared to a cutoff value (2.7132) derived from a logistic regression model that correctly diagnosed > 85% of clinically probable AD participants (Harrington et al., 2013). Three potential participants were excluded either because they were too young to participate or their clinical classification (MCI), resulting in 24 study participants: 10 NATs and 14 PATs. Their CSF Abeta/Tau ratios (mean ± SD) are: CH-NATs (4.95 ± 1.19) and CH-PATs (1.75 ± 0.66). Researchers collected and analyzed EEG data with no knowledge about the group classification. As shown in the previous study, this cohort included two groups of participants that were comparable for age, gender, education, and handedness, as previously described (Arakaki et al., 2019).

### Procedures

Study participants were seated in a quiet room, and were first asked, for resting state baseline measures, to “sit still” and “empty their minds” for 5 min with eyes open (eyes fixed at the letter “E” on the bottom of the dark screen), and then for 5 min with eyes closed.

The brain cognitive challenge, or N-back WM test (*N* = 0, 2 that reflect the load conditions of the task), was administered using E-prime software (Psychology Software Tools, Inc., Sharpsburg PA) on a Dell Precision T5610 with a 20” screen. Procedures were described previously (Arakaki et al., 2018, 2019). Participants were comfortably seated before a computer screen and were instructed, practiced for 2–3 min, and were then tested for 0-back, then for 2-back. We challenged participants’ working memory by visual N-back (identify target letter in a sequential letter presentation), with low load (0-back, identify the target in the presenting letter) and high load (2-back, identify the letter that is the same as two screens back) trials. Each load condition included 3 blocks of 30 trials a block. The N-back task took 12–25 min to complete. As previously reported, neither accuracy (ACC) nor response time (RT) were significantly different between the CH-NAT and CH-PAT participants during the 0-back test; RT during 2-back was not significantly different; ACC for 2-back was significantly better in CH-NAT compared to CH-PATs (Table 1).

**TABLE 1:** Accuracy and reaction time in CH-NATs vs. CH-PATs.

	CH-NAT		CH-PAT		*P*-value	Pooled SD	Cohen’s *d*
	Ave (SD)	Max K-S value*	Ave (SD)	Max K-S value*			
**0-back**							
ACC	0.90 (0.06)	0.17	0.88 (0.06)	0.17	0.441	0.06	−0.33
RT (ms)	574.45 (96.40)	0.1	559.73 (63.23)	0.12	0.679	81.52	−0.18
**2-back**							
ACC	0.82 (0.07)	0.11	0.75 (0.09)	0.15	***0.029***	0.08	−0.87
RT (ms)	865.35 (128.02)	0.19	836.85 (132.36)	0.09	0.602	130.21	−0.22

The mean accuracy (ACC) and mean reaction time (RT) were calculated for both CH-NATs and CH-PATs. There is no significant difference in 0-back for both ACC and RT however, during 2-back CH-NATs performed significantly better than CH-PATs. P-values with < 0.05 are in bold italics to denote significance (Baddeley, 1998b). * K-S values are based on (α = 0.05 critical value of 0.41. SD standard deviation; K-S, Kolmogorov-Smirnov test.

### EEG Recordings

Online EEG data were collected during resting or the WM challenge as previously described (Arakaki et al., 2018). We placed a 21-sensor, dry electrode system (Quasar Wearable Sensing, DSI-24, San Diego, CA) approximately at locations at the international 10–20 system (Fp1, Fp2, F7, F3, Fz, F4, F8, T3, C3, Cz, C4, T4, T5, P3, Pz, P4, T6, O1, O2, M1, and M2). EEG signals were sampled at 300 Hz, and bandpass filtered between 0.003–150 Hz. Three auxiliary sensors were used to record electrooculographic (EOG), electrocardiographic (ECG), and electromyography (EMG) (on the right forearm) activity. A trigger channel encoded the time of presentation of letter stimuli, participants’ responses, and test type (0- or 2-back) for further analysis.

### Behavioral and EEG Data Processing

A researcher collected all behavioral and EEG data and processed them without knowledge of CH-NAT/CH-PAT status. Behavioral performance was described and compared by accuracy (ACC) and response time (RT): ACC was defined as the percentage of correctly responded trials out of total trials; RT as the average duration of time from stimulus onset to participant’s response for correct trials.

We analyzed all data in EEGLAB version 13.4.3b (Delorme and Makeig, 2004) running in MATLAB R2016b (The MathWorks, United States) and custom codes developed in-house. Continuous EEG recordings were segmented into epochs of 2,500 ms duration during eyes closed for resting state or using stimulus onset as a reference during WM, including 500 ms before and 2,500 ms after stimulus onset. Only correctly responded trials were used for analysis because we were interested in activities that are supported by the WM task. Preprocessing steps included epoching, filtering, re-referencing, large artifact removal, and time-frequency analysis. Preprocessing and time-frequency (TF) analyses were as previously described (Arakaki et al., 2018). Briefly, epochs were filtered between 30 and 80 Hz. Epochs with considerable artifact activity greater than three standard deviations (SDs) of each sensor were rejected. For TF analysis, epoched EEG data were decomposed with logarithmic scaling between 30 and 80 Hz by fast Fourier transform and Morlet wavelet [*e*^*i*2π*tf*^*e*^−*t*^2^/2σ^2^^] convolution in the frequency domain, followed by the inverse fast Fourier transform (Cohen and Donner, 2013; Cohen, 2014). Power values were calculated before averaging over epochs. Power values were normalized by decibels to the baseline power from −400 to −100 ms pre-stimulus at each frequency band [$dBpower=10^{*}\text{log}10\left(\frac{power}{baseline}\right)$]. We extracted low gamma (500–1,500 ms, 30–50 Hz) and high gamma (500–1,500 ms, 50–80 Hz) for comparison across sensors, participants, and groups. This was done separately for each sensor, condition, and participant. Gamma power was compared between CH-NATs and CH-PATs. The relationship between gamma power and behavioral performance (ACC and RT) was studied using Pearson’s correlation.

### Statistical Methods

We performed group comparisons on participant baseline characteristics using two-sided *t*-tests or Fisher’s exact test. For each participant, we averaged the total gamma power from all sensors and the gamma power from each sensor for each of the following 6 regions (Lianyang et al., 2016; Arakaki et al., 2018): frontal or F (Fz, F3, F4), central or C (Cz, C3, C4), parietal or P (Pz, P3, P4), left temporal or LT (F7, T3, T5), right temporal or RT (F8, T4, T6), and occipital or O (O1, O2) (demonstrated in the results section). We compared gamma power between two groups (PAT, NAT). As this was an exploratory study, we did not correct for multiple comparisons. Further, since individuals with early AD have demonstrated frontal hyperactivity (Qi et al., 2010; Mormino et al., 2011; Lim et al., 2014; Lopez et al., 2014; Nakamura et al., 2018), we compared gamma power during resting state (eyes open and eyes closed, Supplementary Tables S1, S2), and during the task (0-back, and 2-back) between two groups (CH-NATs and CH-PATs). We used Cohen’s equation $d=\frac{\mu_{1}-\mu_{2}}{S}$ for effect size (ES) to examine the magnitude of difference between two groups (CH-NATs and CH-PATS), where (*d*) is the effect size, μ1 is the control mean, μ2 is the experimental mean, and *S* is the pooled standard deviation. Given that the mean of each population is different, the pooled standard deviation was calculated from pooled variance: $S^{2}=\frac{\left(n-1\right)S_{x}^{2}+\left(m-1\right)S_{y}^{2}}{n+m-2}$, where *S*^2^ is the pooled variance, *n* being the sample size of group 1, *S*_*x*_ is the standard deviation of group 1, *m* being the sample size of group 2, and *S*_*y*_ being the standard deviation of group 2. We compared gamma power for each region between groups using two-sided *t*-tests. We used a Kolmogorov-Smirnov (K-S) test to demonstrate the normality of our dataset and Kendall’s tau to measure correlation in addition to Pearson’s r. We performed all analyses using PRISM v6.07 (GraphPad), MATLAB R2020a, or Excel from Microsoft Office 365. We set a significance level of 0.05 for all tests.

## Results

### Time-Frequency Plots of Mean Gamma During 2-Back

**FIGURE 1:**
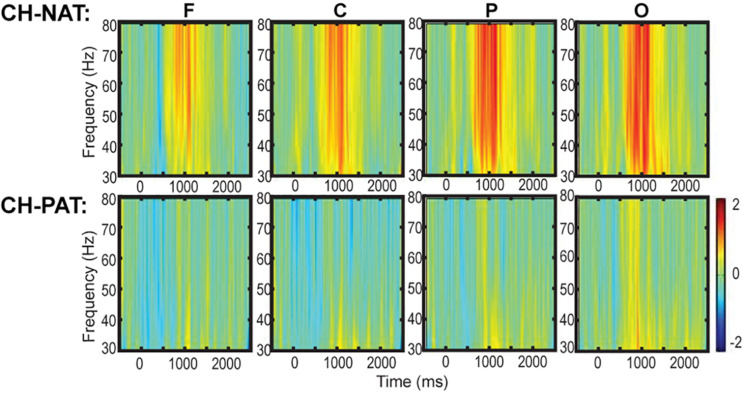
Time-frequency plots (six regions) of mean gamma power during the 2-back test. This is a 3D plot with time reference to stimulus onset (*x*-axis in ms), frequency (*y*-axis in Hz), and power (color scale in dB units) during 2-back testing. Scale bar: power (decibel or dB).

Time-frequency plots of full gamma frequency range (30–80 Hz) during 2-back testing are shown with stimulus onset (ms) by group, in Figure 1. The CH-PAT group tended to have lower gamma across all regions (Figure 1).

### Gamma Power in Brain Regions

**FIGURE 2:**
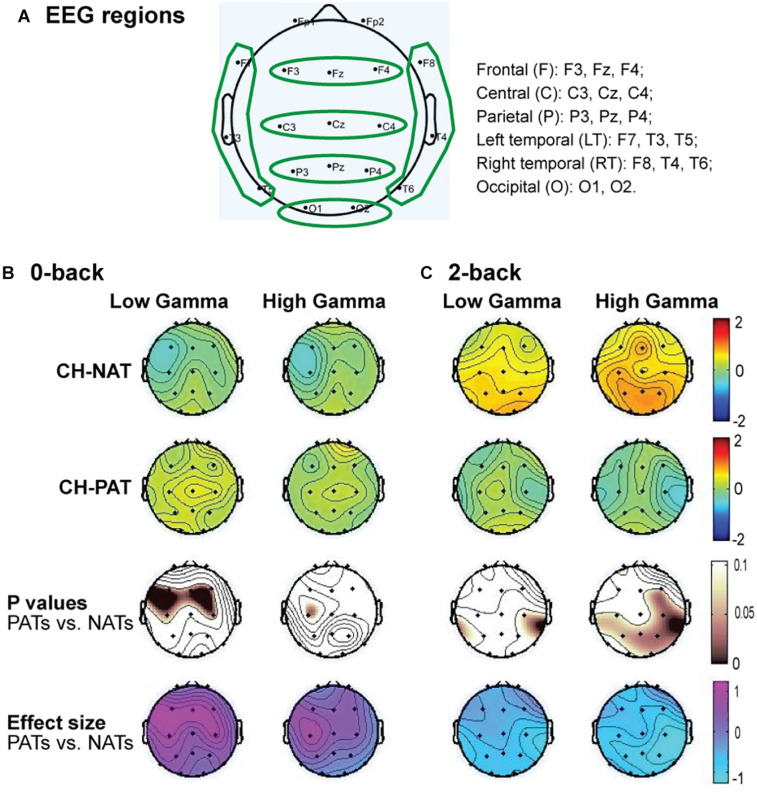
Topoplots of mean gamma power during 0-back and 2-back, by groups. **(A)** Topomap shows groups of EEG sensors for frontal (F), central (C), parietal (P), left temporal (LT), right temporal (RT), and occipital (O) regions. **(B)** Comparisons of low gamma and high gamma power during 0-back testing by groups are shown in topoplots. **(C)** Comparisons of low gamma and high gamma power during 2-back testing by groups. *P*-values and Cohen’s d (effect size) of the between-group differences are shown in the bottom two rows, respectively. During 0-back compared to CH-NATs, CH-PATs have higher low gamma over the frontal region (0.18 ± 0.46 vs. –0.26 ± 0.31, *p* = 0.014, ES = 1.11) and central region (0.36 ± 0.53 vs. –0.15 ± 0.55, *p* = 0.032, ES = 0.95). During 2-back compared to CH-NATs, CH-PATs have decreased low gamma (30–50 Hz) over left temporal region (–0.22 ± 0.63 vs. 0.43 ± 0.72, *p* = 0.045, ES = –0.96), and decreased high gamma (50–80 Hz) over parietal region (–0.06 ± 0.52 vs. 0.90 ± 1.22, *p* = 0.035, ES = –1.02), as shown on Tables 2, 3. Scale bar: power (dB), *p*-value, or Cohen’s *d*.

Figure 2A is a visual representation of the groupings of EEG sensors (F, C, P, LT, RT, and O regions) distributed according to the 10–20 international placement system (Rojas et al., 2018), with some similarity to regions in an earlier study (Dauwels et al., 2010).

Topoplots show different gamma distribution by groups during 0-back (Figure 2B) and 2-back (Figure 2C). During 0-back, compared to CH-NATs, CH-PATs have higher low gamma over the frontal region (0.18 ± 0.46 vs. −0.26 ± 0.31, *p* = 0.014, ES = 1.11) and central region (0.36 ± 0.53 vs. −0.15 ± 0.55, *p* = 0.032, ES = 0.95). Therefore, besides *p*-values, CH-PATs have large, positive ES for greater frontocentral low gamma (ES > 0.80) compared to CH-NATs, and almost no ES for high gamma.

During 2-back compared to CH-NATs, CH-PATs have decreased low gamma (30–50 Hz) over the left temporal region (−0.22 ± 0.63 vs. 0.43 ± 0.72, *p* = 0.045, ES = −0.96), and decreased high gamma (50–80 Hz) over the parietal region (−0.06 ± 0.52 vs. 0.90 ± 1.22, *p* = 0.035, ES = −1.02). Interestingly, in addition to *p*-values, CH-PATs have medium to large negative ES for both low gamma and high gamma compared to CH-NATs, which are homogeneous across the scalp (ES < −0.5).

Details are shown in Tables 2, 3.

**TABLE 2:** Low and high gamma powers for both CH-NATs and CH-PATs during 0-back WM testing.

0-back	NAT			PAT			*P*-value	Pooled SD	Cohen’s *d*
Regions	Ave	SD	Max K-S*	Ave	SD	Max K-S*			
**Low gamma (30–50 Hz)**									
F	−0.26	0.31	0.17	0.18	0.46	0.09	**0.014**	0.41	1.11
C	−0.15	0.55	0.23	0.36	0.53	0.10	**0.032**	0.54	0.95
P	0.01	0.39	0.18	0.24	0.46	0.15	0.221	0.43	0.52
LT	−0.16	0.49	0.14	0.25	0.59	0.10	0.081	0.55	0.76
RT	0.12	0.51	0.17	0.24	1.00	0.16	0.713	0.84	0.15
O	0.13	0.44	0.20	0.31	0.62	0.10	**0.424**	0.55	0.34
**High gamma (50–80 Hz)**									
F	−0.17	0.52	0.26	0.04	0.41	0.08	0.276	0.46	0.46
C	−0.15	0.66	0.25	0.20	0.42	0.10	0.127	0.53	0.66
P	0.02	0.50	0.21	0.09	0.32	0.09	0.697	0.40	0.16
LT	−0.15	0.44	0.21	0.02	0.63	0.15	0.484	0.56	0.29
RT	0.12	0.59	0.13	−0.12	0.57	0.15	0.336	0.58	−0.41
O	0.16	0.42	0.13	0.04	0.48	0.09	0.538	0.46	−0.26

The pooled standard deviation was calculated to increase the precision of the standard deviation between both CH-NATs and CH-PATs. Clinical significance was established using the Effect Size (ES) under Cohen’s Criteria. *K-S values are based on (α = 0.05 critical value of 0.41. SD, standard deviation; K-S, Kolmogorov-Smirnov test.

**TABLE 3:** Low and high gamma powers for both CH-NATs and CH-PATs during 2-back WM testing.

2-back	NAT			PAT			*P*-value	Pooled *SD*	Cohen’s *d*
Regions	Ave	*SD*	Max K-S*	Ave	*SD*	Max K-S*			
**Low gamma (30–50 Hz)**									
F	0.37	1.02	0.11	−0.06	0.60	0.20	0.270	0.84	−0.51
C	0.58	0.99	0.11	0.00	0.55	0.22	0.121	0.80	−0.73
P	0.66	0.96	0.17	0.11	0.49	0.16	0.120	0.76	−0.73
LT	0.43	0.72	0.15	−0.22	0.63	0.25	**0.045**	0.68	−0.96
RT	0.58	0.76	0.14	0.10	0.91	0.17	0.214	0.84	−0.58
O	0.78	0.83	0.15	0.31	0.39	0.16	0.122	0.65	−0.73
**High gamma (50–80 Hz)**									
F	0.60	1.30	0.16	−0.13	0.57	0.26	0.121	1.00	−0.73
C	0.70	1.24	0.24	−0.22	0.66	0.19	0.053	1.00	−0.93
P	0.90	1.22	0.22	−0.06	0.52	0.14	**0.035**	0.94	−1.02
LT	0.55	0.89	0.12	−0.16	0.59	0.21	0.051	0.76	−0.94
RT	0.62	1.13	0.20	−0.20	0.72	0.17	0.068	0.95	−0.87
O	0.87	1.09	0.18	0.14	0.56	0.17	0.077	0.86	−0.84

The pooled standard deviation was calculated to increase the precision of the standard deviation between both CH-NATs and CH-PATs. Clinical significance was established using Effect Size (ES) under Cohen’s Criteria. * K-S values are based on α = 0.05 critical value of 0.41. SD, standard deviation; K-S, Kolmogorov-Smirnov test.

No differences were found between the two groups during eyes open or eyes closed resting state (Supplementary Tables S1, S2).

### Gamma Power Correlates With Accuracy in CH-NATs

Low gamma during 2-back is positively correlated only in CH-NATs with 0-back accuracy over F, C, P, LT, and RT regions (*r* = 0.69∼0.77, *p* = 0.0098∼0.027); high gamma during 2-back is positively correlated with 0-back accuracy over all regions (*r* = 0.68∼0.78, *p* = 0.007∼0.030). High gamma during 2-back is negatively correlated with 0-back response time over P, RT, and O regions (*r* = −0.70∼−0.66, *p* = 0.025∼0.037). Details are shown in Tables 4, 5.

**TABLE 4:** Correlation of gamma and behavior performances during 0-back.

		CH-NAT				CH-PAT			
		ACC_N0	RT_N0	ACC_N2	RT_N2	ACC_N0	RT_N0	ACC_N2	RT_N2
**N0_p**									
Low Gamma	F	0.916	0.788	0.664	0.536	0.091	0.086	0.733	0.557
	C	0.844	0.494	0.536	0.450	0.239	0.250	0.639	0.630
	P	0.966	0.911	0.863	0.595	0.214	0.208	0.945	0.725
	LT	0.706	0.476	0.557	0.830	0.290	0.459	0.702	0.411
	RT	0.771	0.111	0.665	0.655	0.782	0.114	0.834	0.441
	O	0.973	0.811	0.475	0.960	0.177	0.406	0.720	0.172
High gamma	F	0.541	0.530	0.403	0.744	0.089	0.285	0.306	0.981
	C	0.823	0.833	0.572	0.995	0.128	0.713	0.356	0.402
	P	0.908	0.795	0.712	0.901	0.051	0.474	0.944	0.490
	LT	0.630	0.758	0.444	0.970	0.101	0.707	0.665	0.813
	RT	0.374	0.278	***0.036***	0.420	0.422	0.162	0.947	0.830
	O	0.493	0.638	0.726	0.745	0.055	0.633	0.603	0.460
**N0_r**									
Low Gamma	F	−0.04	−0.10	−0.16	0.22	0.56	−0.57	−0.12	0.21
	C	−0.07	−0.25	−0.22	0.27	0.41	−0.40	−0.17	0.17
	P	0.02	−0.04	−0.06	0.19	0.43	−0.44	0.03	0.13
	LT	−0.14	0.26	−0.21	0.08	0.37	−0.26	0.14	0.29
	RT	0.11	−0.54	−0.16	−0.16	0.10	−0.53	0.08	−0.28
	O	0.01	−0.09	−0.26	0.02	0.46	−0.30	−0.13	0.47
High gamma	F	−0.22	−0.23	−0.30	−0.12	0.57	−0.38	−0.36	0.01
	C	−0.08	−0.08	−0.20	0.00	0.52	−0.13	−0.33	0.30
	P	0.04	−0.09	−0.13	−0.05	0.63	−0.26	−0.03	0.25
	LT	−0.17	−0.11	−0.27	−0.01	0.55	−0.14	0.16	0.09
	RT	−0.32	−0.38	−0.66	−0.29	0.29	−0.48	−0.02	−0.08
	O	0.25	−0.17	−0.13	−0.12	0.62	−0.17	−0.19	0.26

*Correlations between EEG gamma power with accuracy (ACC) and response time (RT) for both CH-PATs and CH-NATs during 0-back testing for both low and high gamma power. P-values are reported (in bold italics when < 0.05) and Pearson Correlation “r” (underlined when < −0.5 or > 0.5).*

**TABLE 5:** Correlation of gamma and behavior performances during 2-back.

		CH-NAT				CH-PAT			
		ACC_N0	RT_N0	ACC_N2	RT_N2	ACC_N0	RT_N0	ACC_N2	RT_N2
**N2_p**									
Low Gamma	F	***0.0098***	0.100	0.102	0.449	0.744	0.830	0.451	0.886
	C	***0.016***	0.072	0.136	0.452	0.390	0.640	0.911	0.305
	P	***0.027***	0.059	0.206	0.449	0.713	0.924	0.809	0.535
	LT	***0.024***	0.194	0.150	0.388	0.645	0.779	0.190	0.354
	RT	***0.024***	0.057	0.135	0.546	0.109	0.372	0.739	0.073
	O	0.063	0.052	0.340	0.525	0.743	0.796	0.566	0.555
High gamma	F	***0.017***	0.060	0.090	0.723	0.479	0.992	0.288	0.633
	C	***0.021***	0.088	0.087	0.684	0.764	0.975	0.558	0.531
	P	***0.026***	***0.025***	0.141	0.738	0.965	0.835	0.613	0.777
	LT	***0.007***	0.093	0.054	0.590	0.828	0.963	0.263	0.383
	RT	***0.030***	***0.037***	0.144	0.979	0.097	0.416	0.628	0.196
	O	***0.022***	***0.034***	0.164	0.895	0.786	0.757	0.734	0.724
**N2_r**									
Low Gamma	F	0.77	−0.55	0.55	0.27	−0.12	−0.08	−0.27	−0.05
	C	0.73	−0.59	0.51	0.27	−0.31	0.17	0.04	−0.36
	P	0.69	−0.61	0.44	0.27	−0.13	−0.03	−0.09	−0.22
	LT	0.70	−0.45	0.49	0.31	0.17	−0.10	−0.45	0.33
	RT	0.70	−0.62	0.51	0.22	−0.54	−0.32	−0.12	−0.59
	O	0.61	−0.63	0.34	0.23	−0.12	0.09	−0.21	0.21
High gamma	F	0.73	−0.61	0.56	0.13	−0.25	0.00	−0.37	−0.17
	C	0.71	−0.57	0.57	0.15	−0.11	−0.01	−0.21	−0.23
	P	0.69	−0.70	0.50	0.12	−0.02	−0.08	−0.18	−0.10
	LT	0.78	−0.56	0.62	0.19	0.08	−0.02	−0.39	0.31
	RT	0.68	−0.66	0.50	−0.01	−0.55	−0.29	−0.18	−0.45
	O	0.71	−0.67	0.48	0.05	0.10	0.11	−0.12	0.13

*Correlations between EEG gamma power with accuracy (ACC) and response time (RT) for both CH-PATs and CH-NATs during 0-back testing for both low and high gamma power. P-values are reported (in bold italics when < 0.05) and Pearson Correlation “r” (underlined when < −0.5 or > 0.5).*

Because of the small sample size, we also correlated gamma power with ACC and RT using Kendall’s tau with similar results (Supplementary Tables S3, S4).

## Discussion

In our exploratory study we propose that CH-PATs present more gamma power during the low load condition and less gamma during the high load condition compared to CH-NATs, indicating hyperactivity during low load and insufficient activity during high load. The load difference is consistent with previous findings of altered alpha power in the CH-PATs (Arakaki et al., 2019, 2020). We reported higher risk for cognitive decline for CH-PATs in a longitudinal follow-up study: after 4 years, none of the CH-NATs had declined cognitively, however, 11/28 CH-PATs, or nearly 40% of the group, declined cognitively ranging from significantly impaired to clinically probable AD dementia (Wilder et al., 2018; Harrington et al., 2019). Our follow-up study shows relatively high sensitivity and low specificity. Similarly in a longitudinal study for patients with MCI, Hansson et al. have demonstrated that CSF amyloid and/or tau concentrations have predictive value for progression to AD, where CSF measures show limited specificity (Hansson et al., 2006). Positron emission tomography (PET) imaging may be appropriate for this cause because of higher specificity (Muller et al., 2019). We are planning more extensive follow-up study to address this limitation.

We also show that gamma power correlates with behavioral performance in CH-NATs, but not CH-PATs. First, we noticed only in CH-NATs, accuracy on the 0-back condition was associated with low gamma across F, P, LT, and RT, and high gamma activity in all regions on the 2-back WM condition. This result is consistent with the findings by Stevenson et al. that there is an association between high gamma power and accuracy on a spatial memory task in epileptic patients with an implanted electrode in the dorsolateral prefrontal cortex (DLPFC) (Stevenson et al., 2018). Our results support our hypothesis that CH-PATs have insufficient brain resources for gamma power in the frontal lobe, the center for executive functions, and diminished capability to hold the testing goal compared to CH-NATs.

### Gamma Power Studies in Other Settings

There has been considerable interest in the role that the gamma band plays in cognitive processes. Human and animal studies reveal how gamma is propagated and we present hypotheses of its functionality, summarized in Supplementary Table S5.

#### N-back Working Memory in Relation to Gamma Oscillations

Working memory and other higher-order cognitive processes are optimal when neural oscillations within the gamma frequency bands are synchronized (Bartos et al., 2007; van Vugt et al., 2010; Chen et al., 2014; Cohen, 2014). Since proper modulation of inhibition via GABAergic networks plays a significant role in the generation of gamma oscillations, we hypothesized that CH-PAT individuals may have impaired inhibition mechanisms resulting in hyperactivity during subsequent low load conditions and failed activation in high load condition due to overtaxing (Bartos et al., 2007; Jia and Kohn, 2011; Chen et al., 2014; Palop and Mucke, 2016; Arakaki et al., 2018).

Working memory has been used in various ways as a cognitive task to evaluate the temporary storage and manipulation of information (Baddeley, 1998a; Owen et al., 2005). Previous studies have used working memory paradigms to evaluate cognitive functions, specifically in the frontal cortex. The n-back test is widely used as a reliable measure of WM in the DLPFC (Jonides et al., 1993; Goldman-Rakic, 1994; Courtney et al., 1998; Owen et al., 2005). Furthermore, studies using fMRI during n-back WM show robust activation of several cortical regions, including the lateral premotor cortex, dorsal cingulate and medial premotor cortex, dorsolateral and ventrolateral prefrontal cortex, frontal poles, and medial and lateral posterior parietal cortex (Owen et al., 2005). In a wide variety of cognitive tasks, the parietal cortex is typically involved in the implementation of stimulus response mapping (Kimberg et al., 2000; Miller and Cohen, 2001; Rushworth et al., 2001a, b; Corbetta et al., 2002; Shulman et al., 2002a, b; Andersen and Buneo, 2003; Buneo et al., 2003; Dreher and Grafman, 2003). Studies have also shown a strong association with gamma oscillations and short-term memory, where individuals required to hold information within their working memory showed increased gamma power (Tallon-Baudry et al., 1998; Tallon-Baudry and Bertrand, 1999). Gamma oscillations measured within the neocortex via EEG provide a unique perspective on neuropsychological changes that occur within aging participants who are at high risk of developing AD. In our pilot study, we report gamma power changes occurring within CH-PATs compared to CH-NATs within both the frontal and parietal regions, where CH-PATs show increased frontocentral low gamma power and decreased high gamma in the parietal region during the 2-back condition. Cohen’s *d* analysis suggests there is a global negative effect size in CH-PATs during 2-back. Further validation of these findings will provide insights into changes occurring within these WM regions in high risk AD individuals. N-back WM paradigms have also revealed behavioral changes associated with aging. Several studies have found a strong association between aging and reaction time, with increase in reaction time with age (Gajewski et al., 2018). Furthermore, increased age has been associated with implicated attention and accuracy on n-back working memory tests (Gajewski et al., 2018). Within our cohort we observe that CH-PATs have decreased accuracy in the 2-back condition compared to CH-NATs. Further studies on the association among gamma power, RT, and ACC may provide insights into how gamma oscillations are involved with ACC and RT. Our EEG findings are consistent with Barr et al. (2014) who conducted EEG experiments on cognitively healthy individuals ages 19–60: while our cohort is > 60 years, our results are consistent with their findings of increased gamma activity during high load relative to low load conditions. N-back testing may be a useful tool for observing neurocognitive changes in aging individuals and may provide insight into how gamma oscillations are implicated in the early stages of AD.

#### Gamma Oscillations in Relation to AD Pathology

Gamma oscillations have recently been the focus of several AD studies for their potential therapeutic properties. Recent studies have reported that gamma oscillations are impaired in AD patients and AD animal models, specifically in circuits pertaining to memory acquisition and retrieval (Nakazono et al., 2018). Studies on transgenic AD mice have shown gamma impairments in neuronal spike activity and LFP oscillatory activity. For instance, in a study performed by Goutagny et al. (2013), 1-month old TgCRND8 transgenic mice showed impaired theta-gamma cross-frequency coherence before plaque formation. In terms of tau formation, Booth et al. (2016) showed that in amice model of tauopathy (rTg4510), within the medial entorhinal cortex, gamma activities in dorsal region was preferentially disrupted while those in ventral regions were comparatively preserved. They conclude that this disruptions and the corresponding flattened dorsoventral gradients of theta-gamma coupling may contribute to spatial learning and memory deficit observed in this tauopathy mouse model (Booth et al., 2016). Furthermore, Iaccarino et al. (2016) and Nakazono et al. (2018), using 5XFAD transgenic AD mice models showed impaired gamma oscillations within the hippocampus at 3 months before plaque deposition. In addition, photostimulation of hippocampal circuits within the gamma band increased Aβ_42_ clearance and p-tau reduction, specifically in CA1 (Iaccarino et al., 2016). This suggests that gamma power helps hippocampal fidelity with increased clearance protecting neurons in the memory circuit. In the neocortex, gamma oscillations are impaired within the parietal cortex of J20 AD mice models (Verret et al., 2012; Verret, 2012). Together, these studies demonstrate that the gamma rhythm is impaired in the hippocampus and possibly other regions of the neocortex in AD mouse models before Aβ plaque pathology. Given these findings, qEEG may be a useful tool for detecting gamma power impairments in mice before plaque deposition. Further exploration of the mechanisms underlying gamma impairment is needed. Further studies in humans to validate the use of qEEG as an effective tool for detecting early dysfunction in high-risk AD individuals are needed.

Due to the novelty of the role of gamma power within the brain, few studies have explored gamma power impairments in AD patients. Nonetheless, it is a rapidly expanding field of study that may yield useful therapies for AD. EEG studies have previously characterized AD patients as exhibiting high delta and theta power while showing decreased alpha and beta power (Babiloni et al., 2016a,b,c, d; Wang et al., 2017). Interestingly, Wang et al. (2017) compared resting EEGs between CH and AD patients with both eyes open and closed and showed increased gamma power in AD patients compared to controls (Wang et al., 2017). They propose that the presence of abnormally greater ongoing resting gamma power might be a result of GABAergic interneuron dysfunction within neuronal networks in AD patients; the over couplings between frequency domain may suggest more cognitive resources needed in AD patients to maintain the resting brain state (Wang et al., 2017). In our study, CH-PATs showed elevated gamma power in the low load condition and compromised gamma power in the high load conditions, indicating that possible disinhibition due to disruptions within these networks results in the expenditure of maximum neural resources to maintain the testing goal at low load challenge. Further studies with a larger cohort may further support the use of EEG for early AD detection and validate our hypothesis.

### Mechanisms, Models, and Theories About the Gamma Frequency

The precision timing of neuronal-spiking activities is theorized to play a critical role in the coding of information (O’Keefe and Recce, 1993; Wang and Buzsaki, 1996; Singer, 1999; Buzsaki and Wang, 2012; Buzsaki and Schomburg, 2015). This precision spiking is thought to contribute significantly to the generation of the gamma frequency (Jia and Kohn, 2011). Previous studies have also shown that some cortical neurons show a “resonance phenomenon” at specific frequencies, particularly 10, 20, 40, and 80 Hz (Herrmann, 2001; Herrmann and Demiralp, 2005). One theory for the presence of these resonance frequencies is that neuronal clusters in feedback circuits, such as the visual circuit, transmit time-delayed information from higher to lower processing centers resulting in temporal synchronization of activity at 40 Hz (Herrmann, 2001; Herrmann and Demiralp, 2005). We have not found this specific 40 Hz effect in our data. Future studies are needed to clarify this resonance phenomenon in our aging population. However, even parvalbumin-positive (PV+) cells exhibiting no resonating frequencies exhibit gamma, indicating that timing plays a critical part in the synchronized spiking observed in the gamma rhythm (Fuchs et al., 2007).

Gamma activity is thought to be mediated by GABAergic interneurons firing at specific time points within a spike cycle, increasing the probability of excitatory synchronization in neuronal clusters (Hasenstaub et al., 2005; Jia and Kohn, 2011). This mechanism, GABAergic interneurons firing specifically at the minima of a spike cycle in neuronal clusters within complex circuits, is thought to be associated with gamma activity production and cortical fidelity (Missonnier et al., 2004; Barr et al., 2014). Current research presents several models for the mechanism and propagation of gamma power mediated by GABAergic interactions. One model posits a “stripped-down” network consisting of only inhibitory neurons known as the Inhibitory network gamma (ING) (Wang and Buzsaki, 1996; Buzsaki and Wang, 2012; Buzsaki and Schomburg, 2015). In this model, experimenters noticed that gamma oscillations emerged in two different ways. First, if the firing rate is relatively tonic, there is well-defined periodicity within the gamma rhythm (Kopell et al., 2000). However, stochastic inputs and irregular firing create an unstable asynchronous state that results in gamma emergence (Wang and Buzsaki, 1996; Geisler et al., 2005; Ardid et al., 2010; Buzsaki and Wang, 2012). Synchronization occurs when a cluster of interneurons firing synchronously, creating a spike in the post synaptic neuron during hyperpolarization decay, reinitiates the spike cycle. In this model inhibitory-inhibitory interaction is the driving mechanism for gamma propagation. Another model posits reciprocal connections between excitatory pyramidal and inhibitory interneurons known as the pyramidal-interneuron network gamma (PING) (Wilson and Cowan, 1972; Wang and Buzsaki, 1996; Kopell et al., 2000; Geisler et al., 2005; Buzsaki and Wang, 2012; Buzsaki and Schomburg, 2015). The PING network model describes the delay in pyramidal and intraneuronal spikes and that timing delay is thought to be one of the most prominent features of gamma propagation *in vitro* and *in vivo* (Bragin et al., 1995; Csicsvari et al., 2003; Hasenstaub et al., 2005; Hajos and Paulsen, 2009; Tiesinga and Sejnowski, 2009; Buzsaki and Schomburg, 2015). Studies have shown that the genetic knockdown of AMPA receptors on fast-spiking interneurons reduces the amplitude of the gamma rhythm (Fuchs et al., 2007). Between the two models, the PING model has more support than the ING model as studies show that disconnecting many INGs within the CA1 of the hippocampus does not significantly affect gamma power in mice (Wulff et al., 2009). However, this does not discount INGs since they have been shown to produce gamma oscillations as previously mentioned and gamma is present in regions such as the basal ganglia that possess few excitatory networks. These models are likely cooperative, independent of inhibition or excitation but on the timing of GABAergic neurons within their respective clusters. In our study, we speculate that these networks are compromised within CH-PATs, given their CSF classifications, because of interference caused by abnormal β-amyloid and tau. Previous studies have shown that soluble β-amyloid within the hippocampus of mice causes hyperactivity prior to the formation of plaques (Busche et al., 2012), and a tauopathy mice model shows abnormal gamma activities (Booth et al., 2016). The interference caused by soluble β-amyloid/tau may disrupt the time-specific firing of GABAergic interneurons within INGs and PINGs, resulting in compromised gamma activity, observed in CH-PATs compared to CH-NATs.

### Excessive Low Gamma During Low Load WM and Insufficient Gamma During High Load WM Challenge in CH-PATs vs. CH-NATs

In our study, we evaluated the synaptic mechanisms underlying gamma activity in a cognitive healthy (CH) aging cohort who have been classified by CSF amyloid/tau ratio as either normal (CH-NATs) or pathological (CH-PATs). We show that during low load WM challenge, CH-PATs had increased low gamma activity in the frontal and central regions known to be centers for executive function and higher order processes, suggesting hyper-activity during low load challenge (Baddeley, 1998b). Given the significantly decreased high gamma during the high load challenge, our data may suggest that the high load condition may be overtaxing for CH-PATs, resulting in failing gamma modulation. This condition is consistent with our previous analysis of alpha event-related desynchronization (ERD), where we showed a higher load WM challenge overtaxed CH-PAT participants (Arakaki et al., 2019). Alpha ERD reflects cortical activation (Klimesch, 2012). There are recent reports on the roles that high frequency and low-frequency gamma play in cognition (Ray and Maunsell, 2011). Two leading hypotheses are proposed: first, high gamma functions the same as low gamma oscillations as it pertains to cognition and somatosensory integration, just at a higher frequency and shorter timescale; second hypothesis states that high-gamma power is related to spiking activity unrelated to information processing and integration (Ray and Maunsell, 2011). A study on gamma power and spiking activity in the primary visual cortex (V1) of awake monkeys while varying stimulus size found that low gamma power was anti-correlated with high-gamma power, suggesting that the two phenomena are distinct and have different origins and functions (Ray and Maunsell, 2011). In addition, when baseline power is set as a response measure for bars moving in random patterns on a screen, only bands within the low gamma region (35–45 Hz) showed a significant change in response to the stimulus, showing an increase in spectral power (Sederberg et al., 2007). These findings suggest that low gamma power is a more accurate indication of held attention and learning. However, future studies should explore the role of both high and low gamma in cortical regions.

### Data Interpretation: Gamma Power

During the resting state (both during eyes open and eyes closed), there were no gamma differences between the two groups. Interestingly, there were gamma changes during tasks in CH-PATs versus CH-NATs, and these changes are notably different between 0-back and 2-back. For 0-back, there is almost no effect size in the high gamma band, and a strong, positive, localized (frontocentral) effect size in the low gamma band. Conversely, for 2-back, we observed medium to large negative effect sizes for both low and high gamma bands which are homogeneous across the scalp, based on Cohen’s effect size interpretation (Sawilowsky, 2009). These results are strikingly different between conditions. These data suggest brain hyperactivity during low load challenge, with inefficiency during high load challenge in this early AD stage. This explanation is consistent with alpha power findings in the same population and in other early AD studies (Nava-Mesa et al., 2014; Arakaki et al., 2019). This small preliminary cohort has low statistical power: we only observed significant *p*-values at LT for low gamma and P for high gamma during 2-back testing. However, when adding effect size, we observed a global increase of gamma power based on Cohen’s *d*, which improved our comprehension of this study (Sullivan and Feinn, 2012).

Using a simple WM paradigm, we challenged and unmasked disrupted gamma activity within several cortical regions analogous to treadmill electrocardiogram testing to unmask latent coronary ischemia. Consistent with our previous report on alpha ERD, our exploratory gamma measures from the brain challenge test also has predictive potential for CH-PATs (Arakaki et al., 2019). Our pilot findings encourage further insightful investigations into the possible physiological changes that occur before the onset of AD.

There are negative gamma power values (Tables 2, 3), suggesting lower gamma power than baseline measures. That is not surprising when we average across epochs after calculating power, which gives stronger baseline values than averaging before power calculation (Luck, 2014).

High-frequency brain activity in the gamma range (30–80 Hz) and above overlaps with muscle activity (20–300 Hz), which is difficult to discriminate by a single technique (Muthukumaraswamy, 2013). In this study, we believe the gamma effect is more brain activity than muscle for the following reasons: (1) we ensure data collection has minimal muscle artifact by directing participants to relax the face/neck/shoulder and make sure no visible muscle noise is recorded; (2) in the topoplots, we observed frontocentral low gamma change during 0-back, which is not the edge of the electrode montage (peripheral sensors) and thus less likely to be muscle activity; (3) muscle artifact tends to be higher frequency, such as posterior head muscles peak over 80 Hz (Kumar et al., 2003a, b) and extraocular muscles over 60 Hz (Carl et al., 2012), which tend to contaminate peripheral sensors. Low gamma range activity can be from the frontal facial muscles and, if so, will be mainly at the front sensors (Fp1/Fp2), which did not show changes in the topomap; (4) There are no differences between CH-NATs and CH-PATs during resting state, both eyes open and eyes closed. Therefore, our pilot findings of gamma changes during low and high load WM challenge are more likely to have a brain origin, though they may not be completely free of muscle artifact. Advanced approaches that we did not use to remove gamma, including ICA (Olbrich et al., 2011), beamforming (Hipp et al., 2011), and additional EMG sensors on the face, etc., have downsides, including inter-observer differences and signal complicity.

### Gamma Power Does Not Correlate With Response Time or Accuracy in CH-PATs

Given how little is known about the early stage of amyloid/tau changes in CH-PATs, we show several associations between physiological and behavioral domains in CH-NATs, but not in CH-PATs. That is in line with previous studies on gamma and behavioral performance. Studies in humans have shown that gamma power is correlated with memory retrieval success and modulating accuracy (Schneider et al., 2008; Stevenson et al., 2018). In addition, gamma power is associated with increased congruence in a cross-modality test involving visual and auditory stimuli (Schneider et al., 2008). This association helps us better understand the role gamma power may play in cortical processing, specifically in core executive functions such as WM. Also, our data may provide pilot information for further differentiating how pathological versus healthy aging affects the brain. By measuring gamma power and behavioral components such as accuracy to a standardized baseline in CH individuals, we may be able to unmask physiological dysfunctions that are currently undetected within the elderly population. Future studies that explore the causal relationship between gamma power and dysfunction in both physiological and behavioral domains will allow us to better understand this early stage of AD.

### Limitations and Future Directions

There are some limitations in our study, mainly because it was exploratory, with a relatively small number of participants. Therefore, the small preliminary cohort limited the statistical power of this study, which provides only pilot results that need further investigation. We assessed a highly homogenous cohort, most being Caucasian females of European descent. Future studies should be sex balanced and include a more comprehensive range of participants from different racial and ethnic backgrounds. Nevertheless, both CH-NATs and CH-PATs were age and sex-matched with no significance between the two groups, indicating that these variables did not skew our results. Another limitation is that this was a cross-sectional study. Investigating the relationships between gamma oscillations and CH-PATs longitudinally may help to unmask further neurophysiological dysfunctions underlying pathological versus healthy aging. Our study shows high temporal resolution using qEEG to detect dysfunctions with accurate temporal precision. However, future studies that explore dysfunction in CH-PATs with high spatial accuracy may help elucidate which regions are affected. EEG recordings can often become unrepresentative due to muscle artifacts. Muscular activities significantly contaminate EEG signals complicating further analysis (Chen et al., 2016). Nevertheless, EEG signals can also become unrepresentative during the preprocessing stage (Vorobyov and Cichocki, 2002). For instance, the reference electrode is an extraneous variable that affects the signal (Junghofer et al., 1999). We used widely accepted pre-processing methods to reduce distortions in the signal (Delorme and Makeig, 2004). Although beyond the scope of the current analysis, future studies should implement a standardized method of selecting the reference electrode or using multichannel references to decrease electrode bias (Yao, 2001; Yao et al., 2005; Chen et al., 2016). Finally, further study of the relationship between gamma and low-frequency band power (such as alpha and theta) in CH-PATs may show associations between gamma and lower frequency dysfunction. To further evaluate whether these findings have clinical significance, we are planning a more extensive follow-up study to see if our findings are replicable in a new cohort.

## Conclusion

The objective of this exploratory cross-sectional study was to evaluate and detect potentially compromised gamma activity in CH-PATs compared to CH-NATs using a simple WM paradigm combined with qEEG. The study revealed that gamma activities are compromised in CH-PAT. The results support our hypothesis by showing compromised gamma power in CH-PATs with loss of their gamma correlation with behavioral performance. Our study suggests that further development of WM testing combined with non-invasive qEEG is a possible complementary component of the armamentarium for differentiating early dementia from normal aging.

## Data Availability Statement

The raw data supporting the conclusions of this article will be made available by the authors, without undue reservation, to any qualified researcher.

## Ethics Statement

The studies involving human participants were reviewed and approved by the Huntington Medical Research Institutes (HMRI) Institutional Review Board (IRB). The patients/participants provided their written informed consent to participate in this study.

## Author Contributions

XA and MH conceived and designed the experiments and performed the experiments. RR, XA, QL, AF, and MH analyzed the data. RR and XA wrote the manuscript. RR, QL, AF, MH, and XA edited the manuscript. All authors contributed toward the final manuscript.

## Conflict of Interest

The authors declare that the research was conducted in the absence of any commercial or financial relationships that could be construed as a potential conflict of interest.
