# Supplementary material for: Compromised Behavior and Gamma Power During Working Memory in Cognitively Healthy Individuals With Abnormal CSF Amyloid/Tau
Source: Front Aging Neurosci. 2020 Oct 14;12:574214. doi: 10.3389/fnagi.2020.574214 (PMC7591805; doi:10.3389/fnagi.2020.574214)
Supplement: Supplementary file 1 [file Table_1.docx]

| **Table S1**. Low and high gamma powers for both CH-NATs and CH-PATs during eyes open (EO) resting state. The pooled standard deviation was calculated to increase the precision of the standard deviation between both CH-NATs and CH-PATs. Clinical significance was established using Effect Size (ES) under Cohen’s Criteria. | | | | | | | |
| --- | --- | --- | --- | --- | --- | --- | --- |
| EO | Low Gamma (30-50 Hz) | | | | | | |
|  | NAT | | PAT | | P-value | Pooled SD | Cohen's *d* |
| Regions | Ave | SD | Ave | SD |  |  |  |
| F | -0.15 | 0.57 | -0.09 | 0.64 | 0.797 | 0.61 | 0.11 |
| C | -0.38 | 0.92 | -0.09 | 0.61 | 0.366 | 0.76 | 0.38 |
| P | -0.17 | 0.60 | -0.09 | 0.58 | 0.750 | 0.59 | 0.13 |
| LT | 0.05 | 0.91 | 0.15 | 0.43 | 0.736 | 0.67 | 0.14 |
| RT | -0.02 | 0.33 | -0.14 | 0.58 | 0.562 | 0.50 | -0.24 |
| O | -0.14 | 0.42 | -0.08 | 0.53 | 0.771 | 0.49 | 0.12 |
| EO | High Gamma (50-80 Hz) | | | | | | |
|  | NAT | | PAT | | P-value | Pooled SD | Cohen's *d* |
| Regions | Ave | SD | Ave | SD |  |  |  |
| F | -0.15 | 0.55 | -0.06 | 0.62 | 0.711 | 0.60 | 0.16 |
| C | -0.42 | 1.01 | -0.08 | 0.63 | 0.314 | 0.80 | 0.43 |
| P | -0.20 | 0.64 | -0.08 | 0.60 | 0.639 | 0.62 | 0.20 |
| LT | 0.03 | 0.93 | 0.17 | 0.43 | 0.624 | 0.68 | 0.21 |
| RT | -0.03 | 0.34 | -0.13 | 0.59 | 0.642 | 0.50 | -0.20 |
| O | -0.16 | 0.46 | -0.07 | 0.52 | 0.666 | 0.50 | 0.18 |

| **Table S2**. Low and high gamma powers for both CH-NATs and CH-PATs during eyes closed (EC) resting state. The pooled standard deviation was calculated to increase the precision of the standard deviation between both CH-NATs and CH-PATs. Clinical significance was established using Effect Size (ES) under Cohen’s Criteria. | | | | | | | | |
| --- | --- | --- | --- | --- | --- | --- | --- | --- |
| EC | Low Gamma (30-50 Hz) | | | | | | |  |
|  | NAT | | PAT | | P-value | Pooled SD | Cohen's *d* |  |
| Regions | Ave | SD | Ave | SD |  |  |  |  |
| F | 0.00 | 0.52 | -0.01 | 0.37 | 0.942 | 0.44 | -0.03 |  |
| C | -0.07 | 0.98 | -0.02 | 0.34 | 0.873 | 0.68 | 0.07 |  |
| P | -0.11 | 0.81 | 0.00 | 0.32 | 0.633 | 0.57 | 0.20 |  |
| LT | -0.02 | 0.37 | -0.10 | 0.32 | 0.576 | 0.34 | -0.24 |  |
| RT | -0.04 | 0.43 | 0.02 | 0.36 | 0.694 | 0.39 | 0.17 |  |
| O | -0.08 | 0.56 | -0.16 | 0.50 | 0.722 | 0.52 | -0.15 |  |
| EC | High Gamma (50-80 Hz) | | | | | | |  |
|  | NAT | | PAT | | P-value | Pooled SD | Cohen's *d* |  |
| Regions | Ave | SD | Ave | SD |  |  |  |  |
| F | 0.02 | 0.51 | -0.02 | 0.36 | 0.829 | 0.43 | -0.09 |  |
| C | -0.09 | 1.02 | -0.03 | 0.33 | 0.830 | 0.70 | 0.09 |  |
| P | -0.12 | 0.80 | 0.01 | 0.30 | 0.592 | 0.56 | 0.23 |  |
| LT | -0.01 | 0.36 | -0.09 | 0.31 | 0.562 | 0.33 | -0.24 |  |
| RT | -0.03 | 0.42 | 0.03 | 0.37 | 0.698 | 0.39 | 0.16 |  |
| O | -0.07 | 0.58 | -0.14 | 0.48 | 0.758 | 0.53 | -0.13 |  |

| **Table S3**. Kendall Correlation of gamma and behavior performances during 0-back. Correlations between EEG gamma power during 0-back testing with accuracy (ACC) and response time (RT) for both CH-PATs and CH-NATs. P values are reported (in bold italics when <0.05) and Kendall’s τ (underlined when <-0.5 or >0.5). | | | | | | | | | | |
| --- | --- | --- | --- | --- | --- | --- | --- | --- | --- | --- |
| **N0_p** | | **CH-NAT** | | | |  | **CH-PAT** | | | |
|  |  | ACC_N0 | RT_N0 | ACC_N2 | RT_N2 |  | ACC_N0 | RT_N0 | ACC_N2 | RT_N2 |
| Low Gamma | F | 1.000 | 1.000 | 0.601 | 0.381 |  | 0.073 | 0.108 | 1.000 | 0.727 |
|  | C | 1.000 | 0.381 | 1.000 | 0.108 |  | 0.073 | 0.381 | 0.291 | 0.727 |
|  | P | 1.000 | 0.601 | 1.000 | 0.216 |  | 0.216 | 0.156 | 0.862 | 0.727 |
|  | LT | 0.862 | 0.484 | 0.862 | 0.601 |  | 0.381 | 0.484 | 0.727 | 0.216 |
|  | RT | 0.727 | 0.108 | 0.381 | 0.862 |  | 0.381 | 0.291 | 0.862 | 0.862 |
|  | O | 0.727 | 0.381 | 0.216 | 1.000 |  | 0.108 | 0.484 | 0.862 | 0.216 |
| High Gamma | F | 0.601 | 0.108 | 0.216 | 0.727 |  | 0.108 | 0.291 | 0.601 | 0.727 |
|  | C | 0.862 | 0.484 | 0.291 | 0.862 |  | 0.156 | 0.862 | 0.484 | 0.484 |
|  | P | 0.484 | 0.862 | 0.601 | 0.484 |  | 0.073 | 0.601 | 1.000 | 0.862 |
|  | LT | 0.862 | 0.484 | 0.484 | 0.727 |  | 0.381 | 1.000 | 1.000 | 1.000 |
|  | RT | 0.484 | 0.291 | ***0.029*** | 1.000 |  | 0.381 | 0.291 | 1.000 | 0.727 |
|  | O | 0.291 | 0.381 | 0.601 | 0.727 |  | 0.073 | 0.601 | 0.484 | 0.727 |
| **N0_τ** | | **CH-NAT** | | | |  | **CH-PAT** | | | |
|  |  | ACC_N0 | RT_N0 | ACC_N2 | RT_N2 |  | ACC_N0 | RT_N0 | ACC_N2 | RT_N2 |
| Low Gamma | F | 0.02 | 0.02 | -0.16 | 0.24 |  | 0.47 | -0.42 | -0.02 | 0.11 |
|  | C | 0.02 | -0.24 | 0.02 | 0.42 |  | 0.47 | -0.24 | -0.29 | 0.11 |
|  | P | 0.02 | -0.16 | 0.02 | 0.33 |  | 0.33 | -0.38 | -0.07 | -0.11 |
|  | LT | -0.07 | 0.20 | -0.07 | 0.16 |  | 0.24 | -0.20 | 0.11 | 0.33 |
|  | RT | 0.11 | -0.42 | -0.24 | 0.07 |  | 0.24 | -0.29 | -0.07 | 0.07 |
|  | O | 0.11 | -0.24 | -0.33 | -0.02 |  | 0.42 | -0.20 | -0.07 | 0.33 |
| High Gamma | F | -0.16 | -0.42 | -0.33 | -0.11 |  | 0.42 | -0.29 | -0.16 | -0.11 |
|  | C | 0.07 | -0.20 | -0.29 | -0.07 |  | 0.38 | -0.07 | -0.20 | 0.20 |
|  | P | 0.20 | -0.07 | -0.16 | -0.20 |  | 0.47 | -0.16 | -0.02 | -0.07 |
|  | LT | 0.07 | -0.20 | -0.20 | 0.11 |  | 0.24 | -0.02 | 0.02 | -0.02 |
|  | RT | -0.20 | -0.29 | -0.56 | 0.02 |  | 0.24 | -0.29 | 0.02 | -0.11 |
|  | O | 0.29 | -0.24 | -0.16 | -0.11 |  | 0.47 | -0.16 | -0.20 | 0.11 |

| **Table S4**. Kendall Correlation of gamma and behavior performances during 2-back. Correlations between EEG gamma power during 2-back testing with accuracy (ACC) and response time (RT) for both CH-PATs and CH-NATs. P values are reported (in bold italics when <0.05) and Kendall’s τ (underlined when <-0.5 or >0.5). | | | | | | | | | | |
| --- | --- | --- | --- | --- | --- | --- | --- | --- | --- | --- |
| **N2_p** | | **CH-NAT** | | | |  | **CH-PAT** | | | |
|  |  | ACC_N0 | RT_N0 | ACC_N2 | RT_N2 |  | ACC_N0 | RT_N0 | ACC_N2 | RT_N2 |
| Low Gamma | F | ***0.0466*** | 0.156 | 0.216 | 0.727 |  | 1.000 | 0.862 | 0.484 | 0.727 |
|  | C | ***0.005*** | 0.156 | 0.381 | 1.000 |  | 1.000 | 0.601 | 0.727 | 1.000 |
|  | P | ***0.009*** | 0.108 | 0.484 | 0.862 |  | 0.862 | 1.000 | 1.000 | 0.727 |
|  | LT | 0.073 | 0.381 | 0.727 | 0.601 |  | 0.862 | 0.727 | 0.216 | 0.381 |
|  | RT | ***0.047*** | 0.156 | 0.381 | 0.727 |  | 0.216 | 0.381 | 1.000 | 0.216 |
|  | O | ***0.047*** | 0.291 | 0.381 | 0.727 |  | 1.000 | 1.000 | 0.862 | 0.216 |
| High Gamma | F | ***0.047*** | 0.073 | 0.216 | 1.000 |  | 0.727 | 1.000 | 0.216 | 0.862 |
|  | C | ***0.029*** | 0.108 | 0.156 | 0.862 |  | 0.601 | 0.727 | 0.601 | 1.000 |
|  | P | ***0.017*** | ***0.009*** | 0.381 | 0.862 |  | 1.000 | 0.601 | 0.484 | 1.000 |
|  | LT | ***0.029*** | 0.108 | 0.291 | 0.862 |  | 0.862 | 1.000 | 0.216 | 0.381 |
|  | RT | 0.156 | ***0.005*** | 0.727 | 0.291 |  | 0.291 | 0.291 | 0.601 | 0.727 |
|  | O | 0.073 | ***0.047*** | 0.484 | 0.727 |  | 0.862 | 0.862 | 0.601 | 0.601 |
| **N2_τ** | | **CH-NAT** | | | |  | **CH-PAT** | | | |
|  |  | ACC_N0 | RT_N0 | ACC_N2 | RT_N2 |  | ACC_N0 | RT_N0 | ACC_N2 | RT_N2 |
| Low Gamma | F | 0.51 | -0.38 | 0.33 | 0.11 |  | 0.02 | -0.07 | -0.20 | 0.11 |
|  | C | 0.69 | -0.38 | 0.24 | 0.02 |  | -0.02 | 0.16 | 0.11 | -0.02 |
|  | P | 0.64 | -0.42 | 0.20 | 0.07 |  | 0.07 | -0.02 | 0.02 | -0.11 |
|  | LT | 0.47 | -0.24 | 0.11 | 0.16 |  | 0.07 | -0.11 | -0.33 | 0.24 |
|  | RT | 0.51 | -0.38 | 0.24 | 0.11 |  | -0.33 | -0.24 | -0.02 | -0.33 |
|  | O | 0.51 | -0.29 | 0.24 | 0.11 |  | -0.02 | -0.02 | -0.07 | 0.33 |
| High Gamma | F | 0.51 | -0.47 | 0.33 | 0.02 |  | -0.11 | -0.02 | -0.33 | 0.07 |
|  | C | 0.56 | -0.42 | 0.38 | 0.07 |  | 0.16 | -0.11 | -0.16 | -0.02 |
|  | P | 0.60 | -0.64 | 0.24 | -0.07 |  | 0.02 | -0.16 | -0.20 | 0.02 |
|  | LT | 0.56 | -0.42 | 0.29 | 0.07 |  | 0.07 | -0.02 | -0.33 | 0.24 |
|  | RT | 0.38 | -0.69 | 0.11 | -0.29 |  | -0.29 | -0.29 | -0.16 | -0.11 |
|  | O | 0.47 | -0.51 | 0.20 | -0.11 |  | 0.07 | 0.07 | -0.16 | 0.16 |

**Supplementary Table S5.** A list of informative primary research articles surrounding the study of gamma activity detailing population studied, the paradigm used, measurements are taken, and findings.

| **Population** | **Paradigm** | **Measurement** | **Findings** | **Ref.** |
| --- | --- | --- | --- | --- |
| **Human (n=39).** 20 females, 19 males with diagnosed drug-resistant epilepsy containing surgically implanted depth electrodes. | **Verbal Memory Task:** the test had 15 or 20 nonrepeatable randomly chosen English or German nouns depending on the participants native language. 21 participants received 20 items and 14 received 15 items. Participants were presented with 9 and 60 lists over 1-4 sessions. After each list was presented, they were instructed to compute simple arithmetic and asked to respond vocally when they knew the answer. Participant answers were recorded. | **fMRI & iEEG:** the signals were sampled at 200, 256, 500, 512, or 1024 Hz. Band pass was filtered .3-70 Hz or .1-100Hz. | Their findings show that **gamma activity plays a role in the encoding of information that is subsequently recalled**. Supported by Increased gamma activity recorded in the hippocampus, left temporal lobe, and the lateral intraparietal cortex during encoding. | (17) |
| **Human (n=12)**. Age 26-40. No further information is provided. | **Visual Stimulation Test:** black bars on a screen are presented for 40ms for baseline condition. For the experimental condition, bars moved downwards for 1s at a 3°/s. All bars were presented in the perifoveal region. | **EEG:** 17 electrodes placed on in the occipital area in a 3x3 array with Fpz as the reference using the 10/20 international system. | Evoked gamma (40Hz) activity shifts topographically within the visual cortex relative to the bar position in the visual field it is presented in. | (25) |
| **Human (n=13).** Mean age: 24. 8 males and 5 females all RT handed. | **Visual Short-Term Memory Task:** Two conditions: 1. memory: 2 stimuli presented 400ms apart with 800ms delay. Participants had to detect whether stimulus 2 matched stimulus 1 (20%). 2. dimming: 1 stimulus is presented but participants had to report whether the fixation cross dimmed (80%) or not. | **EEG:** 17 electrodes around the scalp | Induced gamma-band activity is present in the occipitotemporal and bilateral frontal regions when an individual is required to actively hold a represented object in short-term memory. | (18) |
| **Human (n=10).** 4 males and 6 females. Mean age: 24.5 yrs. None were reported to have any neurological or psychiatric disorders. | **Steady-State Visual Evoked Potential (SSVEP):** Custom manufactured goggles with LEDs in each eyepiece was used. A frequency generator was used to drive the LEDs from 1Hz-100Hz in 1Hz increments. | **EEG:** 19 electrodes mounted on an elastic cap | There is a specific resonance phenomenon to selective frequencies at 10Hz, 20Hz, 40Hz, and 80Hz in the visual cortex. This resonance suggests a plausible explanation for the preferential presence of gamma frequencies (40Hz, 80Hz) in cortical processing. | (74) |
| **Ferretes (vivo= 30 cells, vitro= 55 cells).** | **Inhibitory Postsynaptic Potentials (IPSPs) in Cortical Networks:** cells in vivo and vitro were excited using either DC (direct current) or SEVC (single electrode voltage clamp). Frequency powers were measured for both conditions with functional GABAᴬ receptors and while blocked with picrotoxin. | **Local Field Potential (LFP):** direct recordings in layer 5 of the dorsal prefrontal cortex (DPFC) | Their findings show that GABAergic interneurons play a role in pyramidal cell synchronization by modulating spike timing and precision in cortical networks. This suggests that GABAᴬ receptor-mediated networks are necessary for the generation of higher frequencies (>10Hz) such as beta and gamma. | (76) |
| **Mice:** 5XFAD mice were opto-genetically modified in the CA1 region of the hippocampus. APP/PS1 mice, TauP301S mice, and C57B1/6 WT mice were used. | **Gamma Frequency Stimulation:** WT and 5XFAD mice were optogentically stimulated in CA1 with 40Hz or 8Hz flicker and the visual cortex was stimulated in a dark chamber with a LED bulb. 5 conditions were presented, dark, light, 20Hz, 40Hz, 80Hz flicker. All stimulation was done for 1hr. | **LFP, Immunohistochemistry RT-QPCR, RNAseq, Glial isolation/staining:** direct recordings in CA1 and visual cortex. ELISA was used to determine Amyloid β concentration. RT-qPCR & RNAseq was used to measure the glial response. Staining was used to visualize glial cell mobilization. | Their results showed that optogenetically stimulating interneurons at 40Hz, but not other frequencies reduced Amyloid β concentration within CA1 and VC. In addition, cellular and genomic data show increased glial response and clearance of Amyloid β in those regions. | (20) |
| **Human (n=4).** 3 females, 1 male. Mean age: 45yrs. Presurgical epileptic patients with stereotactically implanted depth electrodes. | **Spatial Memory Task:** 100 objects presented within an invisible circle, participants are asked if found 1=indoors 2=outdoors; then the same objects were presented at the top and the participant has to move the object to its original position when first presented. | **Depth Electrode Recording:** direct recordings from implanted electrodes were localized in the dorsolateral prefrontal cortex (DLPFC) or CA1 of the hippocampus. | Their data show that regions involved in memory encoding and precision (CA1 and DLPFC) exhibiting high gamma power strongly correlate with accuracy during retrieval. | (21) |
| **Human (n=46).** RT handed, Mean = 30.52 ±9.83; 23 males & females. | **N-Back WM Test:** 1-3 back Working Memory test stimuli consisted of black Capital letters presented for 250ms followed by a delay for 3s. | **EEG:** 64 electrode caps with 4 auxiliary sensors around the eye referenced to Cz. Impedance was kept below 5kΩ. | Their findings suggest that evoked gamma oscillations may serve as a useful biomarker for aging. | (27) |
| **Human (n=20).** Mean = 32 ±13; 12 males, 18 females. Post-surgery epileptic patients | **Sternberg (1966) short-term recognition memory of synthetic faces:** Participants receive 1-4 stimuli of faces or letters followed by 3s delay. A probe stimulus is then presented, and participants are tasked to indicate whether it was part of the initial stimuli. The maintenance interval was 0-3s after the offset of the last stimulus. Fifteen alternating blocks consisting of 16 letters and faces were presented. | **Depth Electrode Recording:** direct recordings from implanted electrodes were localized based on clinical considerations. 1454 electrodes distributed across varied brain regions. | They demonstrated gamma power (48 –90 Hz) in the human hippocampus increases with memory load during the retention phase of the task. The increase in gamma power was observed in regions related to memory and attention. | (23) |
